# Full Arch All-on-4 Fixed Implant-Supported Prostheses with 8.5 Years of Follow-Up: A Case Report

**Published:** 2018-07

**Authors:** Hakimeh Siadat, Amirreza Rokn, Elaheh Beyabanaki

**Affiliations:** 1 Professor, Dental Research Center, Dentistry Research Institute, Department of Prosthodontics, Tehran University of Medical Sciences, School of Dentistry, Tehran, Iran; 2 Professor, Dental Implant Research Center, Dentistry Research Institute, Department of Periodontics, Tehran University of Medical Sciences, School of Dentistry, Tehran, Iran; 3 Assistant Professor, Department of Prosthodontics, School of Dentistry, Shahid Beheshti University of Medical Sciences, Tehran, Iran

**Keywords:** Mouth Rehabilitation, Edentulous Jaw, Implant-Supported Dental Prosthesis

## Abstract

Typically, full arch reconstruction of edentulous ridges requires five to 10 dental implants; however, some patients demanding fixed implant-supported prostheses are not able to medically or economically afford complex bone grafts and/or a greater number of implants. These situations could pivot the treatment plan toward the All-on-4 protocol. Nevertheless, due to less implant support in this treatment option, mechanical and biological complications might arise. This article describes the treatment of a fully edentulous patient with two types of fixed implant-supported prostheses on four dental implants along with the following complications.

## INTRODUCTION

Implant-supported prostheses are successful treatment modalities that can be used for single tooth replacement to full mouth rehabilitation. Depending on the number of the implants used in fully edentulous patients, the restoration can be removable or fixed [1,2]. Some of the other factors that determine the type of the prosthesis include the remaining amount of bone, the amount of inter-occlusal space, and the patient’s wishes [1–5]. Fixed implant-supported prostheses usually need five to nine implants in the mandible and six to 10 implants in the maxilla [1,2].

Generally, fixed implant-supported prostheses can be divided into screw-retained and cement-retained prostheses. Each treatment option has several advantages and disadvantages compared to the other [6,7]. The main advantages of cement-retained prostheses are esthetics and the passive fit, while screw-retained prostheses offer easy retrievability [6–9].

Another concept for restoring a fully edentulous arch with a fixed prosthesis is called the All-on-4 protocol. An All-on-4 prosthesis is a screw-retained hybrid prosthesis supported by four dental implants [10]. However, considering the complications of this treatment option, it is advised to choose this treatment plan only when placement of an adequate number of implants is not possible [11].

There is another treatment option that combines both of the merits of the screw- and cement-retained fixed prostheses called the Toronto Bridge or abutment hybrid prosthesis [12]. The Toronto Bridge is basically a screwed-in mesostructure for single or multiple replacements that provides custom-made milled abutments for the cementation of individual crowns [12].

The main advantage of this prosthesis is the ability to correct implant angulations while maintaining the passive fit and esthetics and substituting the lost tissues in severely resorbed ridges [12–14]. This type of fixed implant-supported prosthesis is usually used in cases with increased inter-arch spaces. Excessive inter-arch spaces are commonly seen in patients with a previous history of aggressive periodontitis, long-term tooth loss, and/or long-term wearing of complete dentures [12–14].

This article reports the treatment procedure and complications associated with full mouth rehabilitation using metal-resin hybrid prostheses which lasted for seven years. Subsequently, they were replaced with Toronto prostheses on four dental implants in each jaw with a 1.5-year follow-up.

## CASE REPORT

A 55-year-old female with a history of aggressive periodontitis was referred for full mouth rehabilitation. The remaining teeth had to be removed due to excessive mobility. The patient had been wearing complete dentures since the age of 25.

Despite the considerable amount of bone loss and unwillingness to undergo any major bone graft procedures, the patient demanded fixed prostheses.

**Fig. 1:**
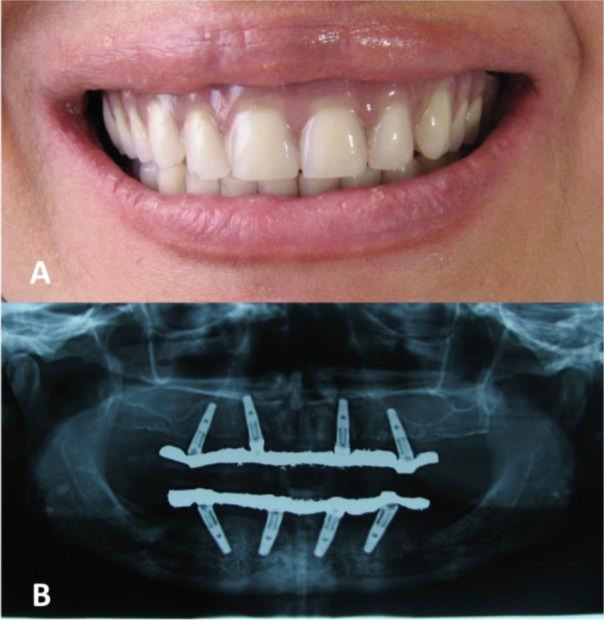
(A) Clinical and (B) radiographic views of metal-resin (hybrid) All-on-4 prostheses

Considering the presented situation, the All-on-4 protocol was selected for full mouth reconstruction. Four regular platform (RP) implants (Replace Select; Nobel Biocare, Göteborg, Sweden) with the diameter of 4.3 mm and the length of 13 mm were inserted in the canine and second premolar areas of each jaw, using the prescribed All-on-4 guide (All-on-4, Nobel Biocare, Göteborg, Sweden). After four months, the uncovery surgery was performed. Two weeks later, the open tray technique with splinted impression copings was used for the impression procedure. After trying in the screw-retained metal framework on multiunit abutments (Nobel Biocare, Göteborg, Sweden) in the mouth and evaluating their passive fit, they were returned to the laboratory for tooth set-up at the previously established vertical dimension of occlusion. After the delivery of the metal-resin (hybrid) prostheses and adjusting the occlusion (Fig. 1. A and B), the patient was followed for complications which mostly were chipping of the pink acrylic resin.

**Fig. 2:**
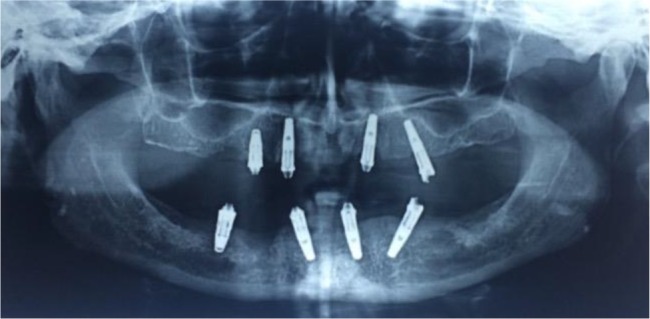
Radiographic view of distal implants replacing the failed previous implants on the left side.

After seven years, the most distal implants on the left side of both jaws had to be removed due to bone loss. Since the patient demanded new and more aesthetically pleasing fixed prostheses, it was decided to replace the failed angulated implants with straight ones (10 mm in length) at a little more distal position (Figure 2).

**Fig. 3:**
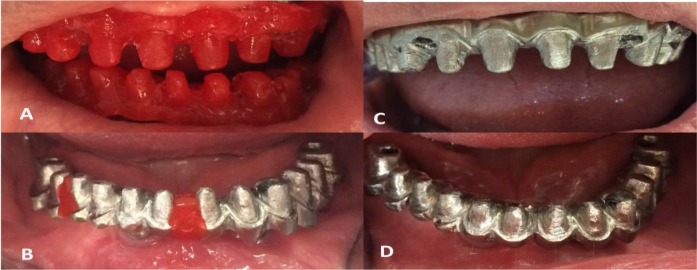
(A) Acrylic resin patterns of mesostructures. (B) The index made for soldering of the mandibular metal mesostructure. (C and D) Parallel individual abutments in metal mesostructures

Since the crown height space in either jaw was more than 15 mm, the Toronto implant-supported prosthesis was chosen as the new treatment plan [12,14]. After impression making using the splinted open tray technique and establishing the vertical dimension of occlusion, multiunit abutments (Nobel Biocare) with proper gingival heights were used. Acrylic resin patterns (GC Corp., Tokyo, Japan) of the mesostructures were fabricated and tried in the mouth to verify the impression accuracy and the passive fit (Fig. 3. A). After casting the mesostructure patterns with nickel-chromium (Ni-Cr) metal alloy (Wiron 99, Bego, Bremen, Germany), they were tried in the mouth. However, despite confirming the accuracy of the acrylic resin patterns, the cutting and soldering of the mandibular metal mesostructure were necessary to achieve the desired passive fit (Fig. 3. B). As with the usual Toronto framework design, parallel individual abutments were made using acrylic pattern resin, and were then cast with metal (Fig 3. C and D).

**Fig. 4:**
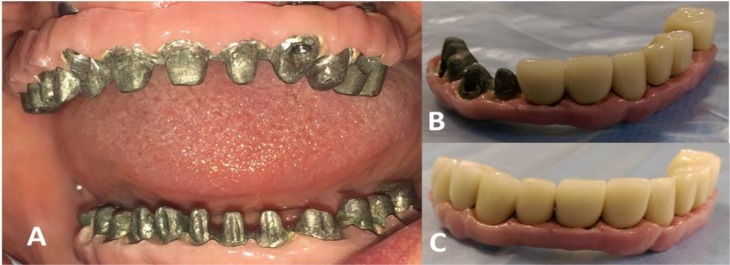
(A) Pink porcelain simulating the lost gingival tissue. (B and C) Individual metal-ceramic crowns adapted on metal frameworks

The modified ridge lap design was used for the tissue side of the frameworks to ensure esthetics and tissue health. Individual metal-ceramic crowns were fabricated using Ni-Cr metal alloy and porcelain (EX3, Kuraray Noritake Dental Inc., Okayama, Japan) with shades of A1 and A2 for cervical and incisal/occlusal halves, respectively. To mimic the gingival tissue color, a pink porcelain (EX3, Kuraray Noritake Dental Inc., Okayama, Japan) was applied to the mesostructures (Fig. 4. A). The crowns (Fig. 4. B and C) were tried in the mouth, and the group function occlusion was used as the occlusal scheme. As the final stage, the screw abutments were fastened in the mouth to 35-newton centimeter (Ncm). Before cementing the crowns, the abutment screw holes were filled with Teflon tape (SITCO, Fujian, China).

**Fig. 5:**
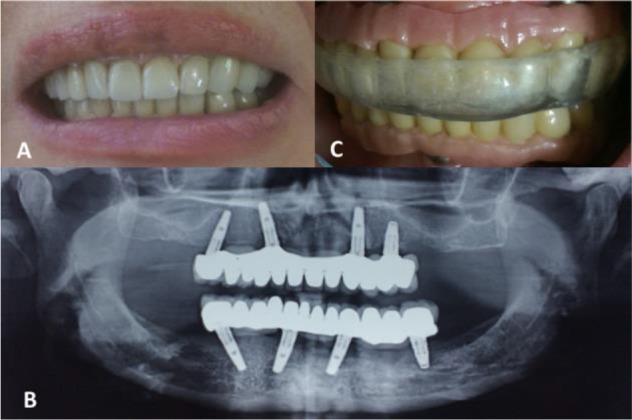
(A) Clinical and (B) radiographic views of metal-ceramic crowns cemented on metal mesostructures. (C) A maxillary night guard for lowering the risk of porcelain fracture

Eugenol-free temporary cement (Temp-Bond^®^ NE, Kerr Corp., Orange, CA, USA) was used for the crowns adjacent to the abutment screw holes [10], while all the other crowns were cemented using zinc phosphate luting agent (Harvard; Harvard Dental International GmbH Margaretenstr, Hoppegarten, Germany; Fig. 5. A and B). A maxillary night guard was also fabricated for the patient to reduce the risk of porcelain fracture by distributing the forces (Fig. 5. C). The patient was instructed to regularly perform oral hygiene measurements including using an interdental brush and super floss as well as the conventional brush and floss. The patient was asked to return for regular follow-ups which were every three months during the first year, and then every six months.

**Fig. 6:**
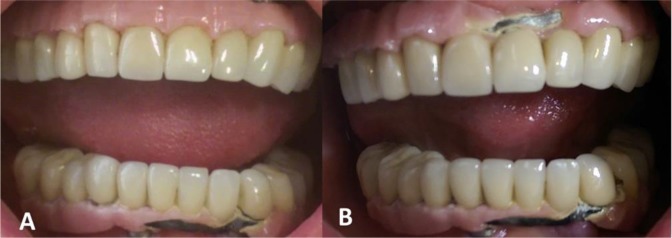
Fracture of the anterior pink porcelain in (A) the lower and (B) upper prostheses after 12 and 18 months, respectively

Twelve months after the delivery of the prostheses, chipping of the pink porcelain was observed in the mandibular anterior region (Fig. 6. A). Also, during the next six months, the same problem happened in the maxillary anterior region (Fig. 6. B). To recover the framework from the mouth, the crowns placed near the abutments’ access holes were removed using a crown remover instrument. After removing the remaining crowns from the mesostructure using the heat from a porcelain oven (Zirkonofen 700 Ultra-Vakuum, Zirkonzahn, Germany), the pink porcelain was applied to the defective parts, and the prostheses were delivered to the patient as described before.

## DISCUSSION

Implant-supported prostheses offer solutions for restoring difficult edentulous situations, which is sometimes impossible via conventional prostheses. Excessive bone loss and alveolar ridge deformities following long-term edentulism and previous periodontitis are among these situations which could restrict the number of implants to four. However, the most common biologic complication in this regard is the failure of at least one implant [15]. Fixed prostheses for the restoration of a fully edentulous arch with four implants could be either made of metal-resin (hybrid) or porcelain-metal structures.

The Toronto prosthesis is usually used when the inter-arch space is too much to be restored with a conventional fixed implant-supported prosthesis [14]. Despite the advantages of the Toronto Bridge, there are some concerns and complications that need to be considered and addressed. When multiple individual crowns are to be tried in the mouth, evaluating the proximal and occlusal contacts can be difficult [14], and it might be needed to use a fit checker material inside the crowns to make them more stable as it was used in this case. Another complication related to this type of prosthesis was achieving and maintaining the required passive fit during the laboratory procedures. Especially when a large one-piece casting framework is considered for restoring a full arch, there is a chance for dimensional changes during the casting process [16]. Also, following firing the gingival pink porcelain (even with a low-fusing porcelain) to the metal substrate, the passive fit of a previously tested framework could be compromised [17]. Therefore, in order to achieve the passive fit, the framework had to be cut and soldered. Using non-engaging multiple unit screw-retained abutments for a one-piece structure or dividing a full arch framework into three segments, particularly when the implant axes hinder a single path of placement, could be considered as the alternative solutions [4]. However, since the bone volume was not sufficient to place the adequate number of implants, in this case, it was not possible to divide the mesostructure into three segments. Furthermore, such segmentation could compromise the final esthetic result due to improper implant number or position in some cases.

Acrylic fracture has been reported to be the most common complication in an All-on-4 prosthesis [15]. This could also be the case when using porcelain in the Toronto Bridge. In both prostheses, this complication could be prevented by regular occlusal adjustments and by using a night guard [18]. The possible reason of acrylic fracture, in this case, was the tension created in the bulky pink porcelain which was used to camouflage the defective and reduced soft/hard tissue structures in some areas in conjunction with using a long span one-piece full arch framework [19–21]. Also, the lack of proprioception capacity of the normal dentition plays a role in the higher incidence of porcelain fracture in implant-supported restorations [20,22]. Pink laboratory composites are also another choice in these situations. However, their drawbacks, in comparison with a pink porcelain, are weaker bonding to the metal substrate and water resorption over time [23].

Considering the later need to access the abutment screw holes, the operator should consider lingual collars at least for the crowns adjacent to the abutment access holes. The collars would prevent chipping of the veneering porcelain when a crown remover instrument is used [24]. Also, it is advisable to reduce the amount of the luting cement to the least needed amount [6], especially for the crowns near the access holes.

Furthermore, the gingival porcelain might inhibit accessibility for complete removal of excess cement; therefore, it is recommended to fabricate duplicated abutments using a putty material or acrylic resin and to cement the crowns on them. After removing the excess cement, the crown can be cemented in the mouth [7]. Cementing the crowns near the abutment access holes with an appropriate temporary luting agent is advisable for their easier future removal.

## CONCLUSION

Less support is provided to counteract the occlusal loading when there are only four implants to support a full arch fixed prosthesis. In these conditions, the All-on-4 prosthesis design might be according to the Toronto Bridge or metal-resin (hybrid) prosthesis. However, regardless of the prosthesis design, careful follow-up is warranted to resolve the associated problems.
